# Comparison of Outcomes Following Knee Resection Arthrodesis using an Intramedullary Nail versus Dual Plating for Tumours About the Knee

**DOI:** 10.5704/MOJ.2511.004

**Published:** 2025-11

**Authors:** DKD Carolino, AR Tud

**Affiliations:** Musculoskeletal Tumor Service (MSTS), Philippine Orthopedic Center, Manila, Philippines

**Keywords:** dual plating, intramedullary nailing, knee resection arthrodesis, Kuntscher nail

## Abstract

**Introduction:**

For extensive osseous involvement of primary tumours in the distal femur and proximal tibia, knee resection arthrodesis (KRA) is an economic alternative to endoprosthetic reconstruction in developing countries. Enneking (1977) described the use of an intramedullary (IM) nail for KRA and is still regarded as the most reliable method for fusion. We sought to determine if dual plating or IM nailing for KRA would produce comparable outcomes.

**Material and Methods:**

This is a cross-sectional study of 30 patients who underwent KRA with either IM nail or dual plates for tumours about the knee. Demographic and surgical profile, functional scores using the Musculoskeletal Tumour Society (MSTS) score, and incidence of complications were determined.

**Results:**

Mean follow-up was 2.28 years (SD 20.4). IM nail was utilised in 12 (40%) and dual plating in 18 (60%). 21 complications occurred, with 11 (52.38%) and 10 (47.62%) occurring in the IM nail and dual plating group respectively. MSTS score was higher with the IM nail (23.5 vs 22.5). Mean operative time was longer with the IM nail compared to dual plating (8.29 vs 7.80 hours). Blood loss was higher with the IM nail (1309.09 vs 1138.89mL).

**Conclusion:**

Outcomes of IM nailing and dual plating KRA are comparable, including the incidence of complications. While blood loss and operative time were noted to be longer in the IM nailing group, and hospital admission was longer in the dual plating group, the difference was not significant. Larger, prospective studies are recommended to report outcomes for fusion done following tumour resection.

## Introduction

Knee resection arthrodesis (KRA) for malignant or benign aggressive tumours was first described by Lexer e*t al* in 1908 as a surgical option for lesions affecting the distal femur or proximal tibia. Enneking *et al* altered the method in 1977 to mitigate complications such as infection, non-union, and fractures associated with the Lexer technique^1^. Various knee arthrodesis techniques have been described since then, each with its own advantages and disadvantages. Incidence of complications ranges from 20% to 85%, and include non-union, bone graft fracture, loss of fixation, palsy, and inadequate soft tissue coverage^2,3^. A lack of consensus remains regarding which fusion method is superior^4^.

The technique of dual plating for knee arthrodesis was introduced by Osgood in 1913, for the treatment of tuberculosis^3,5^. Advantages of this technique include the use of one incision for debridement and implantation in patients with infected arthroplasties, capacity to bridge large osseous defects while maintaining length of the extremity, ease of fixation in the desired position at the time of surgery, and ability to contour plates to accommodate while achieving compression^3,6^. There has since been limited data on the outcomes of knee arthrodesis with dual plates^3^.

Enneking’s KRA technique has been performed at our institution since 1993. The investigators sought to determine if arthrodesis using dual plates following tumour resection about the knee would decrease blood loss and operative time previously associated with the former technique^7,8^, with the goal of producing better short-term outcomes. Socio-economic factors for patients in developing countries also emphasise the need to select more economic options with comparable results.

## Materials and Methods

An analytical cross-sectional comparative study was performed, investigating patients who underwent KRA for malignant and benign aggressive tumours about the knee from 2015 to 2021 at a single tertiary hospital. Techniques compared were dual plating versus intramedullary nailing. All surgeries were performed by a single team of orthopaedic oncologists using either a Kuntscher nail or two standard limited contact dynamic compression plates. Those who did not fulfil these inclusion criteria and/or did not provide consent were excluded in the population.

Purposive data gathering via convenience sampling of hospital records was done using the keywords: “knee”, “resection”, and “arthrodesis”. Patients were filtered according to inclusion and exclusion criteria. Upon identification of selected cases, data on the patients’ admission and operative course were collected retrospectively. For functional outcomes, consenting patients were contacted and advised a scheduled follow-up. Function was rated using the 1993 version of the Musculoskeletal Tumour Society (MSTS) score which evaluated pain, function, emotional, supports, walking, and gait. Approval was obtained from the Institutional Review Board and Ethics Committee prior to commencement of this study.

The general objective of this study was to compare the outcomes of patients diagnosed with benign aggressive or malignant tumours about the knee who underwent KRA using an intramedullary nail or dual plates. Specific objectives are the following, (a) Demographic and surgical profile of the selected patients. (b) Functional outcomes using the MSTS score^9^. Each parameter is scored 0-5 and combined for a possible total score of 35. A score of 23 or greater is considered an excellent result; a score of 15-22 is considered a good result; a score of 8-14 is considered a fair result; and a score of less than 8 is considered a poor result^10^.

(c) Occurrence of complications among participants who underwent KRA with either technique. A complication is defined an event such as wound dehiscence, recurrence, infection, implant failure/loosening, and fracture for which the patient required an intervention.

Sample size was calculated based on the estimation of the population proportion for functional score (MSTS). Assuming that the proportion of post-limb salvage surgery in patients with primary bone tumours with satisfactory results is 92%^11^, with a maximum allowable error of 5%, and a reliability of 80%, the sample size required is 49.

Statistical analysis was performed using the Stata 18 BE. Cross-tabulation of frequencies for characteristics was done between the treatment group for each of the baseline characteristics. The Shapiro-Wilk test was used to determine if the data was normally distributed. T-test was used for comparison of normally distributed data, while the Wilcoxon Rank Sum Test was used for non-normally distributed data. Significance level is set at 0.05 for both comparisons and testing correlations.

## Results

**Fig. 1:**
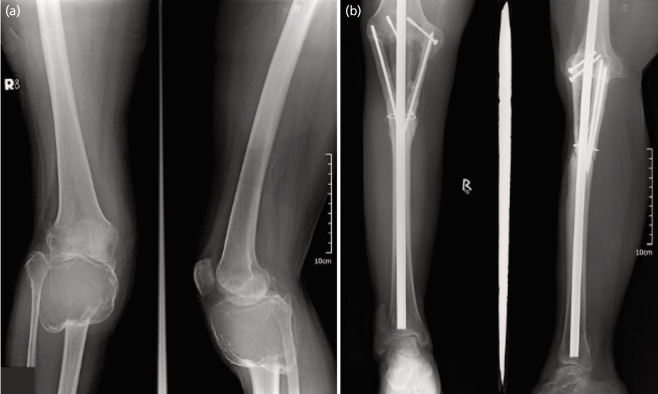
KRA with IM nail in a 51/F. (a) Pre-operative radiographs show a giant cell tumour of the proximal tibia, for which proximal tibia resection and KRA was performed. (b) Three years post-operatively show good union with fibular grafts proximally and distally.

**Fig. 2:**
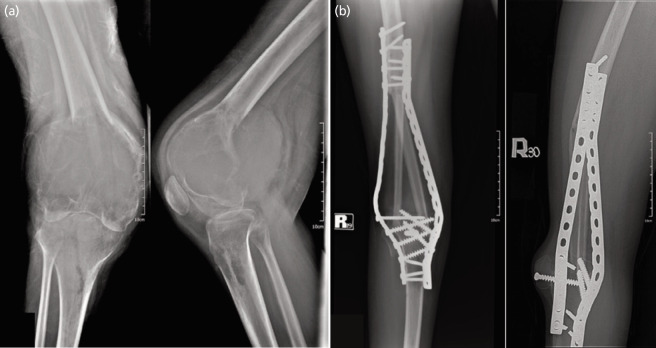
KRA with dual plates in a 24/F. (a) Pre-operative radiographs show a distal femur giant cell tumour with soft tissue extension. Distal femur resection and KRA were performed. (b) The 3.5 years post-operatively show good union with fibular grafts proximally and distally.

Thirty patients diagnosed with benign aggressive and malignant lesions about the knee who underwent KRA were included into the study population. Twelve (40%) underwent arthrodesis with the use of an intramedullary nail, while 18 (60%) of patients underwent arthrodesis with dual plating, all of which were performed by the same team of orthopaedic oncologists. Fig. 1 and 2 demonstrate sample cases of KRA done with the use of an IM nail and dual plate, respectively. Table I illustrates the demographic and surgical characteristics of the study population. The mean age of patients was 38.7 (SD 13.09). Majority of respondents were noted to be female (n=19, 63.33%), with giant cell tumour (GCT) as the most frequent diagnosis (93.33%). The proximal tibia (n=17, 56.67%) was the most common tumour location.

**Table I:** Demographic and surgical characteristics of the population (N=30).

Demographic and Surgical Characteristics of the Population (N=30)	Specifications	Treatment Group		Total (n)	Total (%)
		Dual plate (n=18, 60%)	IM nail (n=12, 40%)		
Sex	Female	10	9	19	63.33
	Male	8	3	11	36.67
Age	30 or less	7	3	10	33.33
	31 to 40	5	4	9	30
	41 to 50	2	1	3	10
	51 to 60	3	3	6	20
	61 or older	1	1	2	6.67
Diagnosis	GCT	16	12	28	93.33
	Malignant	1	0	1	3.33
	Other	1	0	1	3.33
Bone	Distal femur	5	8	13	43.33
	Proximal tibia	13	4	17	56.67
MSTS score	Excellent	9	5	14	46.67
	Good	7	3	10	33.33
	Fair	2	1	3	10
	Poor	0	1	1	3.33
	Unspecified	0	2	2	6.67
Complications	Infection	3	5	8	38.10
	Wound dehiscence	4	2	6	28.57
	Non-union	0	2	2	9.52
	Implant irritation	0	1	1	4.76
	Palsy	2	1	3	14.29
	Implant failure	1	0	1	4.76

Mean follow-up was 2.28 years (SD 20.4), with significant difference in follow-up time between the two treatment groups (p-value 0.0147). Most recent MSTS score showed outcomes that were excellent in 46.67%, good in 33.33%, fair in 10%, and poor in 3.33% of cases. Those who underwent IM nailing had a median MSTS score of 23.5, while dual plating had 22.5.

A total of 21 complications occurred, including eight cases of infection, six cases of wound dehiscence, three cases of peroneal nerve palsy, two cases of non-union, and one case each of implant irritation and implant failure. Eleven complications (52.38%) occurred in the IM nail group while 10 (47.62%) occurred in the dual plating group.

Table II shows the mean and standard deviation (SD) for normally distributed variables as well as the median for non-normally distributed variables. Distribution was determined by the Shapiro-Wilk test. Mean operative time was longer in the IM nail group (mean 8.29 mins, SD 1.49 mins) compared to dual plating (mean 7.80 hours, SD 1.50 hours). Median MSTS score was higher in the IM nail group (23.5 vs 22.5), as was median duration of follow-up compared to the dual plating group (32 vs 12 months). Patients were admitted in the hospital for a longer period in the dual plating group versus the IM nailing group (13.5 vs 10 days). While not significantly different, it is notable to mention that blood loss was higher in the IM nail group (mean 1309.09 vs 1138.89mL). Only the duration of follow-up showed significant difference with a p-value <0.05 (p-value 0.0147) (Table III).

**Table II:** Distribution of variables for comparison.

Treatment Group	Comparison	Mean	SD	Median
Dual Plate	Operative time (minutes)	7.80	1.50	-
	MSTS score	-	-	22.5
	Duration of follow-up (months)	-	-	12
	Length of admission (days)	-	-	13.5
	Blood loss (mL)	1138.89	538.40	-
IM Nail	Operative time (hours)	8.29	1.49	-
	MSTS score	-	-	23.5
	Duration of follow-up (months)	-	-	32
	Length of admission (days)	-	-	10
	Blood loss (mL)	1309.09	364.57	-

**Table III:** Correlation analysis of variables for comparison among the treatment groups.

Comparison	p-value	Difference
Operative time	0.3909	Not significant
MSTS Score	0.9808	Not significant
Duration of follow-up	0.0147	Significant
Days of admission	0.2184	Not significant
Blood loss in mL	0.4004	Not significant
Complications	0.3145	Not significant

*Significant difference at p-value <0.05

Table IV shows the occurrence of complications among the two treatment groups. Dual plating had a total of nine complications, while the IM nail group had eight complications. The p-value of the Fischer’s Exact Test is 0.465. Hence, there was insufficient evidence to confirm a significant association between incidence of complications and treatment group.

**Table IV:** Presence of complications among the treatment groups.

Treatment Group	Without Complications	With Complications
IM nail	4	8
Dual plate	9	9
Total	13	17

## Discussion

The distal femur and the proximal tibia are among the most common sites affected in patients with primary bone tumours^12,13^. Osteosarcoma is one of the most common malignancies of childhood, with an initial peak in the first to second decade of life^14^. Among cases, 75% affect the distal portion of the femur, while 80% affect the proximal portion of the tibia. Giant cell tumour, on the other hand, is also one the most common benign aggressive bone tumours appearing in the third to fourth decades of life. They are likewise found adjacent to the knee, with 40-50% arising from the distal femur and proximal tibia^10,11,15^. Similarly, patients included in our study population with tumours about the knee were predominantly diagnosed with giant cell tumour followed by osteosarcoma, occurring in the first three decades of life.

With extensive tumours about the knee, various reconstruction modalities have been used including arthroplasty, osteo-articular allografts, and arthrodesis^12,13^. However, in developing countries, endoprostheses are costly and large segment allografts are difficult to procure due to lack of immediate availability^12,13^. Hence, KRA has often been the treatment of choice in patients with tumours about the knee due to its low cost, while producing relatively predictable outcomes and enabling a durable limb with limited disability due to loss of motion^3,12,16^.

KRA is performed by extra- or intra-articular resection of the epiphysis and adjacent affected metadiaphyseal area of the proximal femur or proximal tibia with appropriate margins for a specific tumour, followed by reconstruction. The technique was primarily designed by Lexer in 1908 but due to complications such as infection, non-union, and fractures, Enneking evolved the method in 1977 making use of an intramedullary rod and autogenous cortical grafts in an attempt to improve outcomes within the confines of an arthrodesis^1^. Among the 20 patients reviewed, complications include one local recurrence (5%), one wound slough (5%), one pulmonary embolus (5%), one implant failure (5%), two implant irritation (10%), four non-union (20%), four transient peroneal nerve palsies (20%) that resolved spontaneously, and four fibular graft fractures (20%). Four of out seven patients with malignant diagnoses died of disease, while the 16 remaining patients became ambulatory without assistive device^17^. More recent studies show implant failure, non-union (12%), periimplant infection (10-15%), iatrogenic fracture (12%), and implant irritation (12-40%)^8,18^. Similar complications were likewise noted in our study, showing infection (41.67%), wound complications (16.67%), non-union (16.67%), implant irritation (8.3%), and peroneal nerve palsy (8.3%) among patients who underwent KRA using the IM nail.

The technique of dual plating in KRA was first introduced by Osgood in 1913 for the treatment of tuberculosis^5^. The technique has subsequently been adapted for managing severe arthritis secondary to traumatic, degenerative, or autoimmune causes, failed arthroplasty, and tumours^2,6^. Other authors emphasised the advantages of this technique, including utilisation of one incision for debridement and implantation in patients with infected arthroplasties, capability of bridging gaps while maintaining length of the extremity in cases with large osseous defects, fixing it in any desired position at the time of surgery, ability to contour plates in those with significant bony deformities, while achieving compression^3,6^. Despite these advantages, several complications have also been reported with the technique including non-union (22.2%), peri-implant fractures (925%), infection (66%)^3,4,6,19^. Similar complications were also noted in our study, showing infection (16.67%), wound complications (22.2%), palsy (11.1%), and implant failure (5.56%).

Long operative time and significant blood loss is associated with arthrodesis with the use of an intramedullary nail^2,8^. Donley *et al* found an average blood loss of 1,725ml and average operative time of 7.3 hours among patients with tumours, concluding that the long duration and large amount of blood loss are the two drawbacks of this technique^7^. In a more recent study of Crockarell in 2005 of IM nail arthrodesis for failed total knee arthroplasties, they showed quicker average operating time of 210 minutes and an average blood loss of 1,143ml^8^. Newer studies with alterations in the technique show an average range of blood loss of 860 to 2,600ml, and average surgical time of 2.8 hours to 3.5 hours in patients with infection and knee arthroplasty patients^8,18^. The longer time found in our study may be due to the time needed to reconstruct large defects following resection of tumours. Further, Panagiotopolous *et al*^18^ reported a mean hospital stay of 11 days following IM nail arthrodesis. This is similar to the result of our study with a median duration of 10 days. The dual plating group had a longer stay of 13 days, but the difference was not found to be significant (p-value 0.2184). This may be due to longer rehabilitation and gait retraining done while patients remained admitted.

Reported overall fusion rates in literature review have varied from between 50% and 100%. Among the fusion techniques, many surgeons regard the use of the intramedullary nail to be the most reliable in achieving bony fusion. In the recent study of Schwarzkopf, plate fixation arthrodesis has a fusion rate of 81.5% while plating had a fusion rate of 77.8%^4^. In our study, two cases of non-union (9.52%) were reported from the IM nail group, while no cases of non-union were reported in the dual plating group. These results are comparable to other studies^3,4,18^. It must be noted however that there is a significant difference (p-value 0.0147) in the follow-up time of the two groups in our study. This may be due to earlier use of IM nailing for KRA in our institution in 1993, as compared to dual plating which was only initiated in 2018. Longer follow-up periods especially for fusion utilising the dual plating technique is recommended to better determine rates of union.

The main goal of knee arthrodesis is excision of the tumour with appropriate margins, while relieving pain and restoring function and mobility within the limitations of the fusion. The study of Bassiony of IM nail arthrodesis performed in patients with GCT of the distal femur showed functional scores ranging from 20 to 27, with a median score of 23.413. This is similar to outcomes reported in our study, showing a median score of 23.5 among the same population. On the other hand, a study by Saikia *et al* in 2010 also among the same population but utilising a condylar plate showed functional scores ranging 23 to 28, and a median score of 2612. This is higher than the median score of 22.5 in our study, however, further correlation analysis between the MSTS scores of dual plating vs IM nail arthrodesis did not show a significant difference.

At present, the local cost of one long Kuntscher nail and one regular compression plate is similar at an estimated price of 18,000 PHP (325 USD). With dual plating arthrodesis, the projected cost is 36,000 PHP (650 USD). This should be taken into consideration, alongside prolonged operative time and increased blood loss with IM nail arthrodesis, and extended hospital stay in dual plating arthrodesis, despite no significant difference between these parameters and functional outcomes.

It is worth noting that majority of the review of literature reported complications and operative information of KRAs performed for failed total knee arthroplasties. We recommend prospective studies with longer follow-up periods and larger population to more accurately determine complications associated with these procedures solely after distal femur or proximal tibia resection for neoplasms.

## Conclusion

In patients undergoing knee resection arthrodesis for malignant and benign aggressive tumours about the knee, the use of dual plating appears to be comparable to IM nailing in terms of operative time, blood loss, length of hospital stays, occurrence of complications, and functional outcomes. Duration of follow-up was noted to be significantly longer in the IM nailing group; however, this may be due to more recent utilisation of dual plating arthrodesis in our institution. There is lack of research adequately reporting outcomes regarding fusion done for large defects following tumour excision supporting the need for larger prospective studies with longer follow-up. As in all surgeries, we advocate individualised surgical plans catered to the different and specific needs of each patient.
